# DNMT1 regulates polarization of macrophage-induced intervertebral disc degeneration by modulating SIRT6 expression and promoting pyroptosis *in vivo*

**DOI:** 10.18632/aging.204729

**Published:** 2023-05-17

**Authors:** Yang Hou, Jiangang Shi, Yongfei Guo, Guodong Shi

**Affiliations:** 1 Department of Orthopaedic Surgery, Changzheng Hospital, Second Military Medical University, Shanghai 200003, China

**Keywords:** intervertebral disc degeneration, macrophage polarization, DNA methyltransferase 1, sirtuin 6

## Abstract

Background: Intervertebral disc degeneration (IDD) is a complex phenomenon and a multifactorial degenerative disease that creates a heavy economic burden on health systems globally. Currently, there is no specific treatment proven to be effective in reversing and delaying the progression of IDD.

Method: This study consisted of animal and cell culture experiments. The role of DNA methyltransferase 1 (DNMT1) on regulating the M1/M2 macrophages polarization and pyroptosis, as well as its effect on Sirtuin 6 (SIRT6) expression in an IDD rat model and in tert-butyl hydroperoxide (TBHP)-treated nucleus pulposus cells (NPCs) were explored. Rat models were constructed, followed by transfection with lentiviral vector to inhibit DNMT1 or overexpress SIRT6. The NPCs were treated with THP-1-cells conditioned medium, and their pyroptosis, apoptosis, and viability were evaluated. Western blot, histological and immunohistochemistry staining, ELISA, PCR, and flow cytometry were all used to evaluate the role of DNMT1/ SIRT6 on macrophage polarization.

Results: Silencing DNMT1 inhibited apoptosis, the expression of related inflammatory mediators (e.g., iNOS) and inflammatory cytokines (e.g., IL6 and TNF-α). Moreover, silencing DNMT1 significantly inhibited the expression of pyroptosis markers IL- 1β, IL-6, and IL-18 and decreased the NLRP3, ASC, and caspase-1 expression. On the other hand, M2 macrophage specific markers CD163, Arg-1, and MR were overexpressed upon silencing DNMT1 or SIRT6 overexpression. At the same time, silencing DNMT1 exerted a regulatory effect on increasing the SIRT6 expression.

Conclusions: DNMT1 may be a promising potential target for IDD treatment due to its ability to ameliorate the progression of the disease.

## INTRODUCTION

Intervertebral disc degeneration (IDD) is one of the leading causes of disability worldwide. It is a chief contributor to low back pain, which is associated with diminished quality of life and a massive economic burden [1, 2]. Despite its prevalence, the available treatments generally focus only on alleviating symptoms rather than delaying its progression [3].

Previous studies have proposed the potential role of macrophage polarization in the process of IDD [4]. The pro-inflammatory effect of M1-polarized macrophages and the anti-inflammatory effect of M2-polarized macrophages play significant roles in maintaining tissue homeostasis [5, 6]. Previous studies have recognized a remarkable increase in M1-polarized macrophages after disc injuries, which leads to local inflammation and structural changes [7]. Conversely, M2-polarized macrophages had a protective effect on IDD [8]. Hou et al. showed that silencing DNA methyltransferase 1 (DNMT1) has the ability to stimulate the M1-M2 polarization of macrophages, increase the expression of anti-inflammatory agents, and suppress the secretion of inflammatory agents in the IDD process [9].

Furthermore, it has been reported that the Sirtuin 6 gene (SIRT6) can inhibit the secretion of inflammatory agents and mitigate the IDD process. Deficiency of SIRT6 in diabetes promotes macrophage differentiation into the M1-phenotype. In addition, SIRT6 overexpression could induce the differentiation of macrophages into M2 and improve IDD.

As inhibition of DNMT1 could ameliorate the IDD process, SIRT6 has the potential to antagonize the expression of DNMT1 and thereby improve IDD by promoting macrophage polarization. Although M1 polarization of macrophages is associated with pyroptosis in many processes, the effect of M1 polarization of macrophages on the process of pyroptosis in disc degeneration remains unclear. It is also important to note that pyroptosis can be inhibited by SIRT6. Our research includes *in vitro* and *in vivo* investigations that aim to determine the regulatory role of DNMT1 on macrophage polarization and SIRT6 expression in a rat IDD model.

## MATERIALS AND METHODS

### Experimental design

This study consisted of animal and cell culture experiments. Firstly, a randomized controlled animal experiment was conducted on 36 three-month-old male Sprague-Dawley (SD) rats that were obtained from Beijing Huakang Biotechnology Co., Ltd. The experiment was designed and performed according to Changzheng Hospital, Second Military Medical University. The Changzheng Hospital, Second Military Medical University Institutional Animal Center and Biosafety Committee approved the experiment protocol.

Lentivirus plasmid carrying shRNA that targets DNMT1 (lent-sh-dnmt1) to inhibit DNMT1, SIRT6 (lent-p-sirt6), which promotes SIRT6 expression, or a sham injection were injected through the tail vein. Rats were randomly divided into one of the following groups: model group (received no treatment after surgery), model + sh-DNMT1 (inhibited DNMT1), model + sh-NC (negative control shRNA), model + p-SIRT6, model + P-NC (negative control plasmid), and sham surgery group (control). The animals were fed a standard diet and kept in 12-hour light-dark cycle cages. The temperature was set at 25° C and 48% humidity.

### Rat IDD model

Rats allocated for IDD model surgery were anesthetized using inhaled 4% isoflurane. The surgeries were performed under aseptic conditions. An incision was made over the posterior lumbar spine, and the paraspinal musculature was bluntly dissected along the lumbar vertebrae. The interspinous and supraspinous ligaments were excised. The wound was then closed using surgical sutures, and the animal was monitored and moved to the recovery cage. The sham surgery group underwent a posterior midline incision along the lumbar vertebrae and the wound was sutured without any further procedures.

### *In vivo* experiment method

Twelve weeks after surgery, the animals were euthanized using an intraperitoneal overdose injection of 3% pentobarbitone sodium. The harvested tissues were washed using phosphate-buffered saline (PBS) and then preserved at -80° C. The harvested nucleus pulposus tissues selected for hematoxylin and eosin (H&E) staining were fixed with 4% paraformaldehyde (Solebao Technology Co., Ltd., Beijing, China).

### Lentiviral vector construction

The roles of DNMT1 and SIRT6 in disc degeneration were investigated by constructing a lentiviral vector to inhibit DNMT1 or overexpress SIRT6. The DNMT1 (Gene ID: 1786) and SIRT6 (Gene ID: 51548) genes were assembled and inserted into an LV5 lentiviral vector, which was synthesized by transferring BamHI and NotI digested fragments.

One week after IDD surgery, the rats’ tail veins were injected with 3 μL of PBS, which included 0.5 nM of pLKO lentivirus plasmid. The plasmids contained either an shRNA targeting DNMT1 (lent-sh-dnmt1) to inhibit DNMT1 or SIRT6 (lent-p-sirt6), which promotes SIRT6 expression. The control groups were injected with an empty vector lentivirus plasmid.

### H&E staining

The staining protocol was adopted from a previously published study by Li et al. [10]. Briefly, the tissue sections were decalcified using nitric acid in a low-temperature oven and then dehydrated and embedded in paraffin. The area of interest was cut into 5-μm thick sections. The obtained sections were stained with hematoxylin (Jiancheng, Nanjing, China) for 10 minutes and then rinsed with running water. Decolorization of the excess hematoxylin was performed using 1% hydrochloric acid-alcohol for 30 seconds. Next, the slides were immersed in tap water for 15 minutes, and eosin solution (Beyotime, Shanghai, China) was applied for 2 minutes, followed by washing with running water. Finally, the slides were dehydrated and sealed. The slides were observed under an XDS-500C microscope (Shanghai Caikon Optical Instrument Co., Ltd., Shanghai, China).

### Immunohistochemistry staining

The nucleus pulposus tissues were washed three times and fixed with 4% paraformaldehyde for 30 minutes. The tissues were then embedded in paraffin. After sectioning the tissue, xylene and alcohol were used for deparaffinization and rehydration. For permeabilization, 0.1% Triton X-100 diluted with PBS was applied for 20 minutes. Bovine serum albumin (BSA) (3%) and 0.05% Tween-20 were applied for 30 minutes for blocking. Next, the samples were incubated overnight at 4° C with the primary antibody, including anti-NLRP3 (ab263899, Abcam, Cambridge, MA, USA) and anti-caspase-1 (SC-392736, Santa Cruz Biotechnology, Inc., Santa Cruz, CA, USA). After rinsing with PBS, the secondary antibodies were applied for 1 hour at room temperature. Finally, the slides were observed under a microscope (Leica, Wetzlar, Germany).

To quantify the degree of degeneration, a modified grading scale was established according to Hoogendoorn and Boos et al. studies [11, 12]. The score, which ranged from 0 to 8 is based on the following items: anulus fibrosus organization, border integrity between anulus fibrosus and nucleus pulposus, cellularity of the nucleus pulposus, and nucleus pulposus extracellular matrix status. Normal tissue with a score of 0-2.0 was given. A score of 2.1-4.0 represents mild IDD changes. Moderate IDD changes scored 4.1-6.0, and a score of 6.1-8.0 was given when severe IDD changes were presented.

### Nucleus pulposus cells (NPCs) isolation and culture

NPCs were isolated from the euthanized rats. Briefly, several segments of lumber vertebrae were harvested using a surgical blade. The fiber ring was cut using a surgical blade to obtain the nucleus pulposus tissues.

The harvested gel-like tissue was isolated, minced, and digested for 2 hours using 0.25% trypsin-EDTA digestion solution (Solarbio, Beijing, China). The obtained samples were filtered using a 70-μm filter to separate NPCs from tissue aggregates. After rinsing by PBS, the samples were centrifuged at 135g for 3 min, and cultured in Dulbecco’s Modified Eagle Medium culture medium (Invitrogen, Carlsbad, CA, USA) supplemented with 100 μg/mL streptomycin, 100 U/mL penicillin (P1400-100, Solarbio, China), and 10% fetal bovine serum (Gibco, Gaithersburg, MD, USA). When the cells reached 80%-90% confluency, 0.25% trypsin EDTA was used for cell passaging. Culture medium was changed every two days, and the cells which were used in this experiment were between passage one (P1) and passage three (P3).

The resting macrophages (M0) activation was induced by using 100 ng/mL human monocyte cell line (THP-1). Briefly, THP-1 was treated with 100 ng/ml phorbol 12-myristate 13-acetate (PMA) (P1585, Sigma, USA) for 72 hours to achieve macrophage polarization. THP-1 cells were preserved in Roswell Park Memorial Institute (RPMI-1640) media (ATCC Manassas, VA, USA) added to 1% penicillin/streptomycin, 10% fetal bovine serum, and 0.05 mmol/L 2-mercaptoethanol (Sigma Chemical Co., St. Louis, MO, USA), then the monocytes differentiated into macrophage-like cells. Next, the resultant cells were co-cultured with the above-mentioned NPCs for 48 hours. To induce oxidative stress, NPCs were treated with 100 μM TBHP (Sigma-Aldrich, St. Louis, MO, USA) and incubated in the culture medium for 24 hours. Cells were transfected with si-RNA to inhibit DNMT1 or overexpress SIRT6.

### Flow cytometry

For apoptosis detection, NPCs treated with conditional medium from THP1 cells, were centrifuged at 1,000 RPM for four minutes after digestion by 0.25% trypsin and resining three times. The samples were then resuspended in binding buffer till adjusting the density to 1 x 10^6^ cells/mL. The samples were incubated with PI and FITC-Annexin for 15 minutes. Flow cytometry (BD Biosciences, San Jose Diego, CA) was then used to verify cell apoptosis.

### TUNEL detection of nucleus pulposus cells apoptosis

Nucleus pulposus samples from all groups were randomly selected for terminal deoxynucleotidyl transferase dUTP nick-end labeling (TUNEL) detection. The TUNEL apoptosis assay kit (Beyotime, Nanjing, China) was used to label DNA strand breaks enzymatically to assess NPCs apoptosis. Briefly, the nucleus pulposus sections were incubated for ten minutes in H2O2 (3%) at room temperature, followed by rinsing with PBS. After adding 50 ml of TUNEL solution, the sections were incubated for one hour in a humidified condition. Quantification of apoptotic NPCs was performed via counting TUNEL-labeled apoptotic cells.

### Real-time PCR (RT-PCR)

Trizol reagent (15596026, Invitrogen, Carlsbad, CA, USA) was incubated with nucleus pulposus tissue/ NPCs and centrifuged at 12000 RPM for 15 minutes at 4° C. The resultant supernatant was centrifuged again at 12000 RPM for 15 minutes followed by adding 200 μL of chloroform to separate total RNA. The resultant supernatant was added to an equal volume of isopropanol and centrifuged at 12000 RPM for 15 minutes at 4° C, then the supernatant was discarded. Next, 1 mL of 75% absolute ethanol was used to rinse the precipitate and the samples were centrifuged at 4° C, 12000 RPM for 5 minutes, then the supernatant was discarded. Then the samples were kept at room temperature. DEPC water (20 μL) was used to dissolve the RNA.

To reverse mRNA transcription One Step RT-PCR Kit (TaKaRa, Dalian, China) was utilized according to the company’s recommendations. RT-PCR reaction of RNA was performed using a real-time qPCR system (ABI 7300, Applied Biosystems, Foster City, CA, USA). The 2-ΔΔCt calculation method was used to obtain the relative expression values, which were normalized to GAPDH. The test was done three times and the primer sequences were designed by Primer3 V.0.4.0 (Table 1).

**Table 1:** The primers sequences for real-time PCR.

**Gene**		**Primer sequences (5’-3’)**
DNMT1	Forward	AGGACCCAGACAGAGAAGCA
	Reverse	GTACGGGAATGCTGAGTGGT
SIRT6	Forward	GGCTACGTGGATGAGGTGAT
	Reverse	GGGCTTGGGCTTATAGGAAC

### Co-immunoprecipitation (Co-IP) assay

To test the *in vivo* Co-IP, rats were transfected with lentivirus plasmid to evaluate DNMT1 / SIRT6 binding, and IgG was utilized as a control. Next, SDSPAGE was used to separate immunoprecipitated proteins and cell lysates. Then, the samples were transferred to a nitrocellulose membrane, and western blot was used for analysis.

### GST pull down

The p-DNMT1coding sequence was inserted into a lentiviral vector. Glutathione Sepharose 4B beads were used to purify the fusion protein and GST protein as per manufacturer’s recommendations. GST- SIRT6 protein (5 μg) was incubated to cell lysates (500 μg) at 4° C overnight. Next, glutathione Sepharose 4B beads were incubated for four hours with the cell lysates. Then, beads were rinsed three times, and finally loading buffer was used to elute immunoprecipitated proteins.

### Western blotting

NPCs /tissues were lysed using 150-250 μL/20 mg RIPA buffer containing 1% phosphotransferase inhibitor and 1% proteinase inhibitor (R0020, Solarbio Technology Co., Ltd., Beijing, China), centrifuged at 12,000 g at 4° C for 15 minutes and total protein was collected and stored at -80° C. Proteins were quantified using BCA Protein assay kit (PICPI23223; Thermo-Fisher Scientific, Waltham, MA, USA). Then, 180 μL BCA working solution was added to each well. The purification of NPCs’ proteins was performed using gel electrophoresis to transfer the protein into polyvinylidene difluoride (PVDF) membrane (HATF00010, Millipore, Billerica, MA, USA). Proteins were quantified through using a BCA Protein assay kit (PICPI23223; Thermo-Fisher Scientific, Waltham, MA, USA). Then 180 μL BCA working solution was added to each well. The purification of NPCs’ proteins was performed using gel electrophoresis to transfer the protein into PVDF membrane (HATF00010, Millipore, Billerica, MA, USA). A blocking solution consisted of 5% non-fat milk powder diluted in Tris-buffered saline with 0.1% Tween 20 was added at room temperature for two hours. The PVDF membrane was then incubated overnight with the primary antibody at 4° C. Next, Tris-buffered saline with 0.1% Tween 20 solution was used to wash the samples three times (5 minutes each time). After that, the PVDF membrane was incubated for two hours with HRP-labeled secondary antibodies (1:1000) at room temperature, followed by washing three times (5 minutes each time). Finally, the blots were observed in the imaging system using enhanced chemiluminescence (Thermo Fisher Scientific, Waltham, MA, USA).

### Enzyme-linked immunosorbent assay (ELISA)

The harvested tissue was rinsed using normal saline to remove the blood and dried using filter paper, then the tissue was ground and lysed using cell lysate. The samples were then centrifuged at 4° C, 10,000 rpm for 10 minutes, and the supernatant was collected. An appropriate amount of supernatant was collected for protein quantification and ELISA. The ELISA kit (Wuhan AmyJet Scientific Inc., Wuhan, Hubei, China) was used for the determination of IL-6 (No. K4143-100), TNF-α (No. K1051-100), NOS (No. K4169-100), IL-1β (No. K4794), IL-18 (No. K4169-100), Arg-1 (No. K567-100), and CD-163 (No. ab182422). ELISA kit used the double antibody sandwich method to evaluate the level of target protein. The microplate was utilized as solid-phase. The antibody was added to the micropores, then mixed with HRP labeled anti-bodies and undergone protein binding according to the manufacture’s recommendations. After rinsing, the substrate TMB added for color development. Absorbance (OD value) was calculated at 450 nm wavelength using microplate reader (Labsystems, Helsinki, Finland). Moreover, the concentration was obtained through standard curve drawing.

### Viability assay

CCK-8 assay (CP002; SAB Biotech, College Park, MD, USA) was used along with flow cytometry to test NPCs’ viability. A cell suspension of 1-5 × 10^4^ cells/mL was prepared. 100 μL of the solution were cultured in each well of the 96-well culture plate and a 100μl of culture medium was cultured as a control. The cells were incubated at 37° C and 5% CO2 overnight.

At 0 h and 24 h, a conditional medium from THP1 cells was added and incubated for one hour at 37° C. Then, microplate reader was utilized to measure the absorbance at the wavelength 450 nm.

### Transmission electron microscopy

The NPCs were fixed using 2.5% glutaraldehyde solution overnight, followed by treatment with 2% osmium tetroxide. The NPCs were then stained by 2% uranyl acetate and dehydrated using acetone solution, followed by sectioning and staining using toluidine blue. A Hitachi transmission electron microscope was used to capture the images.

### Statistical analysis

Prism 8 (GraphPad Software, San Diego, CA, USA) was used to analyze the results. The outcomes were presented as mean ± SD. t test was adopted for two groups comparison, and one-way ANOVA test was utilized for multiple groups comparison, and a p-value of <0.05 was accepted as statistically significant.

### Availability of data and materials

The data are free access to available upon request.

## RESULTS

### DNMT1 is up-regulated and the expression of SIRT6 is down-regulated in the nucleus pulposus tissue of IDD rats

**Figure 1:**
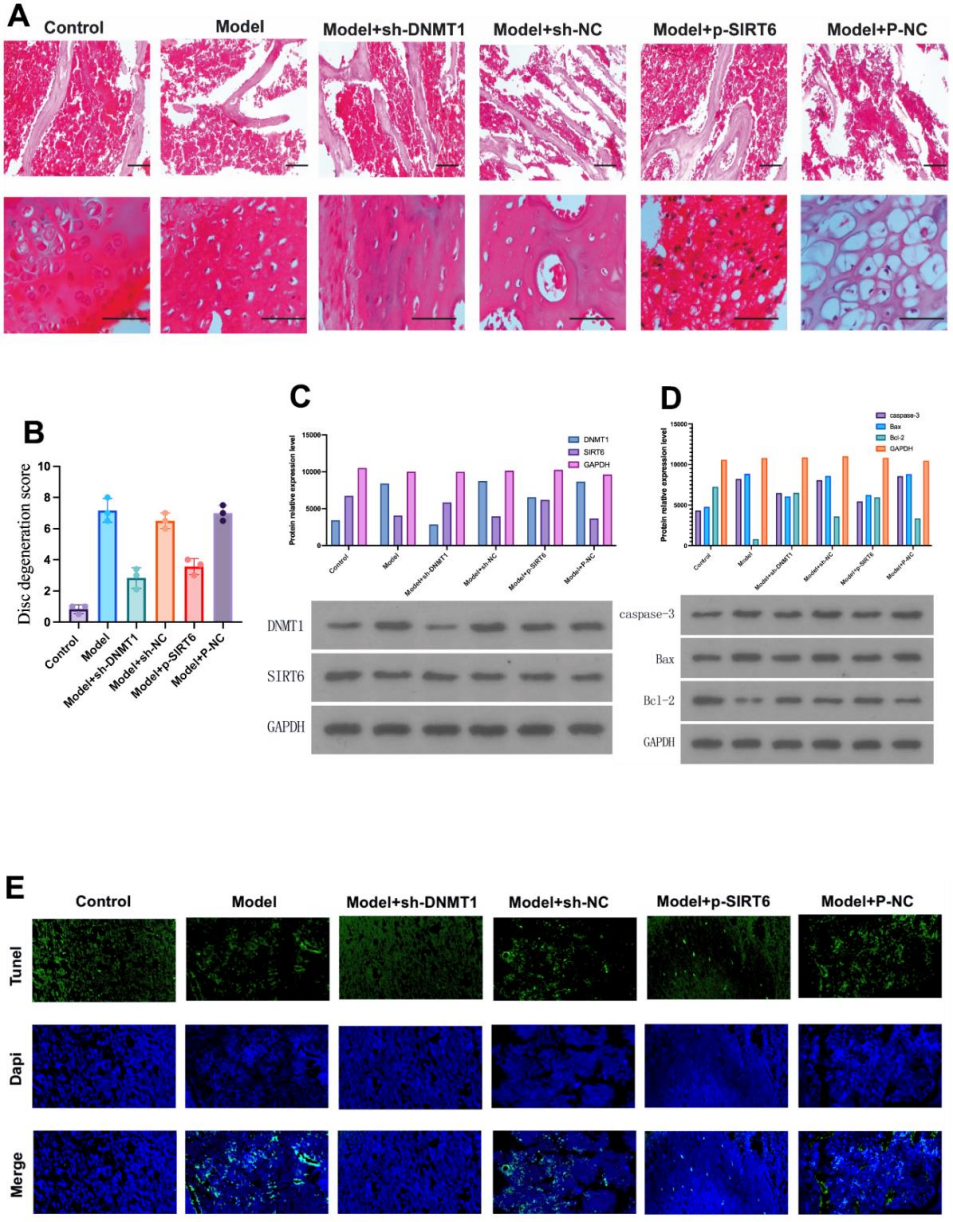
**DNMT1 was up-regulated and the expression of SIRT6 was down-regulated in the nucleus pulposus tissue of IDD rats.** (**A**) Hematoxylin and eosin stain were used to detect the damage to the nucleus pulposus tissue of the rats in each group at 12 weeks. (**B**) Histological damage score significantly increased in the model group and was improved by inhibition of DNMT1 and overexpression of SIRT6 respectively (p<0.05). (**C**) Protein relative expression level of DNMT1 detected by western blot. (**D**) Western blot detection of apoptosis-related proteins including caspase-3, BAX, and BCL-2. DNMT1 inhibition and SIRT6 overexpression significantly inhibited apoptosis p<0.05. (**E**)TUNEL apoptosis assay of nucleus pulposus cells.

Twelve weeks after surgery, HE was used to evaluate the histological condition of the nucleus pulposus of the rats in each group. In the H&E staining, the microscopic observation of the NPCs of intervertebral disc showed a normal finding in the sham surgery group, characterized by an abundance of large and round notochordal cells, and NPCs were arranged in islands (Figure 1A).

On the other hand, the IDD model groups showed distinct degenerative changes. The NPCs were scattered, and the number of notochordal cells was lower than that in the sham surgery group. Moreover, NPCs were replaced by small and long fibroblast-like cells, and round chondrocytes were often observed. However, these degenerative changes were obvious in the model, model + sh-NC, and model + P-NC groups, whereas the model +sh-DNMT1 and model + p-SIRT6 groups were less affected.

These findings are in accordance with the disc degeneration score. The score (mean ± SD) of the sham surgery control group and Model +sh-DNMT1 were 0.8 ± 0.2 and 2.8 ± 0.6, respectively with p<0.05. The score of the model group was the highest 7.1 ± 0.7 indicating severe degeneration changes (Figure 1B). Abovementioned results reveal the role of inhibition of DNMT1 and overexpression of SIRT6 in improving the histological finding.

Western blot was used to detect the expression levels of DNMT1 and SIRT6 in nucleus pulposus tissue. Quantitative results of RT-PCR showed that the model group showed high expression of DNMT1 and low expression of SIRT6. Moreover, sh-DNMT1 significantly reduced DNMT1 expression level and increased SIRT6 expression (p<0.05) (Figure 1C).

The TUNEL assay of specimens obtained 12 weeks after surgery illustrated that the apoptotic cells were significantly increased in the model, model + sh-NC, and model + P-NC groups, while it was lower in the model +sh-DNMT1 and model + p-SIRT6 groups (Figure 1E). These results were in accordance with the western blot analysis which was used to detect apoptosis related proteins including caspase-3, Bax and Bcl-2 (Figure 1D). The abovementioned results suggest that inhibition of DNMT1 and overexpression of SIRT6 could inhibit apoptosis.

### Silencing DNMT1 or overexpressing SIRT6 inhibits M1 polarization of macrophages

**Figure 2:**
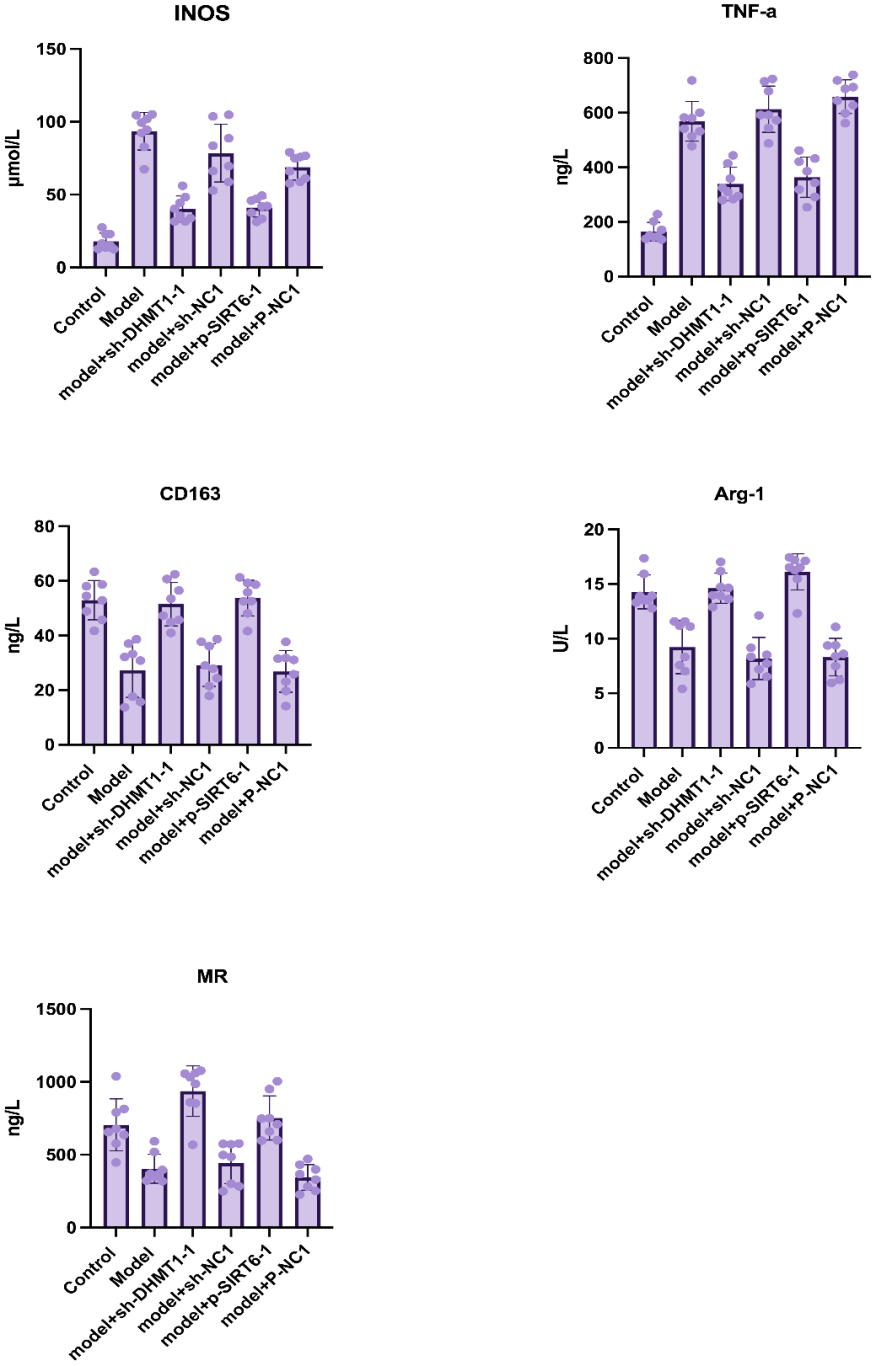
**Macrophage M1/M2 markers in nucleus pulposus tissue homogenate determined by ELISA after 12 weeks of surgery.** Data are presented as the mean and SD. M1 macrophage specific markers: iNOS and TNF-α, and M2 macrophage-specific markers: CD163, Arg-1, MR.

ELISA was used to detect the expression level of macrophage M1/M2 markers in nucleus pulposus tissue fluids. Investigated M1 macrophage-specific markers were iNOS and TNF-α. While CD163, Arg-1, and MR were the studied M2 macrophage-specific markers. The results suggested that inhibition of DNMT1 and overexpression of SIRT6 could significantly decrease the expression of M1 macrophage specific markers and increase the expression of M2 macrophage specific markers, which play a role in nucleus pulposus regeneration (Figure 2).

The experiments were performed for 8 samples of each group, and all markers’ concentrations showed a significant difference when comparing the experimental groups (P<0.001).

### Silencing DNMT1 or overexpressing SIRT6 inhibits the pyroptosis of NPCs in the nucleus pulposus tissue of the IDD model

**Figure 3:**
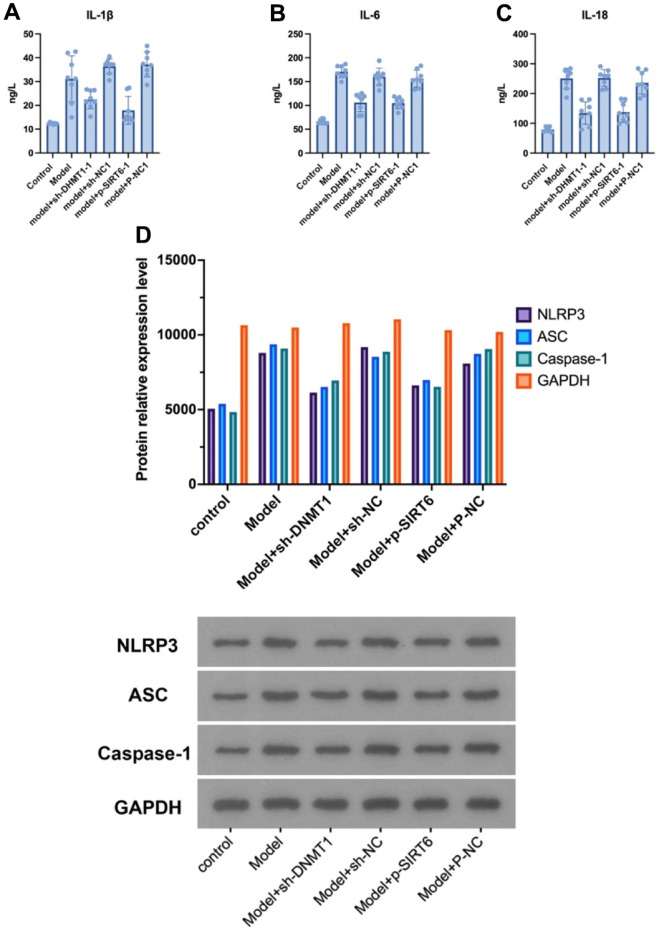
**Silencing DNMT1 or overexpressing SIRT6 can significantly inhibit the NPCs’ pyroptosis.** (**A**–**C**) represent the expression of pyroptosis markers: IL- 1β, IL-6, and IL-18 in the NPCs determined by ELISA. (**D**) Western blotting of NLRP3, ASC and caspase-1 pyroptosis markers.

The NLRP3, ASC and caspase-1 proteins were detected in nucleus pulposus by western blot, and the expression of IL-1β, IL-6, and IL-18 was detected by ELISA to investigate the effect of silencing the DNMT1 or overexpressing SIRT6 on inhibiting the NPCs’ pyroptosis in an inflammatory environment. ELISA results showed that silencing the DNMT1 or overexpressing SIRT6 can significantly inhibit the expression of pyroptosis markers IL-1β, IL-6, and IL-18 (p<0.05), which can accelerate degeneration of the intervertebral disc (Figure 3A–3C). Moreover, in the western blot analysis, the expression of NLRP3, ASC and caspase-1 were significantly decreased in model +sh-DNMT1 and model + p-SIRT6 groups (p<0.05) (Figure 3D).

### DNMT1 in NPCs suppresses M1 polarization of co-cultured macrophages by regulating SIRT6

Macrophages (M0) induced by monocyte THP-1 (induced by PMA 100 ng/mL, 72 h) were co cultured with NPCs to investigate the role of DNMT1 on suppression the M1 polarization of macrophages and regulating SIRT6.

**Figure 4:**
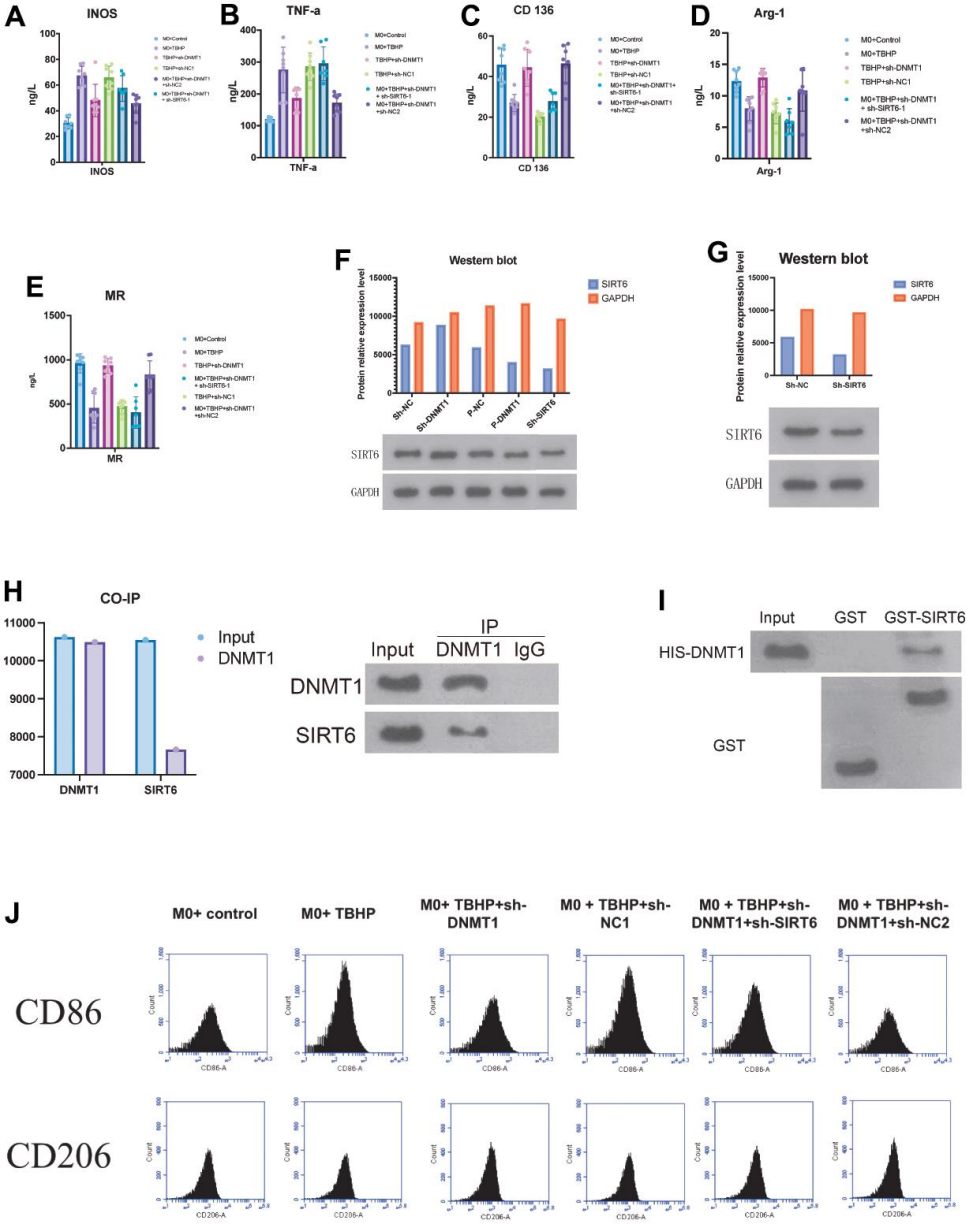
**DNMT1 in NPCs suppresses M1 polarization of co-cultured macrophages by regulating SIRT6.** (**A**–**E**) ELISA detection of the M1/M2 markers expression. (**F**, **G**) negative regulatory role of DNMT1 on SIRT6 expression. (**H**) GST pull-down and CO-IP experiments were used to detect the binding of DNMT1-SIRT6 and the CO-IP experiment of dnmt1-sirt6 interaction. (**I**) GST-pull down assay and immunoprecipitation assay were used to detect the interaction between SIRT6 and DNMT1. (**J**) the CD206 and CD86 for the macrophage phenotypes of all groups were detected by flow cytometry.

For further clarification of the molecular mechanisms, the interaction between DNMT1 and SIRT6 was examined. As exhibited by GST pull-down assay, the interaction between SIRT6 and DNMT1 was detected. GST-pull down assay and immunoprecipitation assay showed that SIRT6 directly interacts with DNMT1 and were important for the transcription activation (Figure 4I).

Moreover, Co-IP experiments also revealed that DNMT1 has an interaction with SIRT6 in NPCs (Figure 4H). The expression of the M1 macrophages specific markers (iNOS and TNF- α) (Figure 4A, 4B), and the expression of M2 macrophage specific markers CD163, Arg-1 and MR were detected by ELISA (Figure 4C–4E). The M1 macrophages markers (iNOS and TNF-α) were presented in a significant low concentration in the sh-DNMT1 and sh-DNMT1+sh-NC2 TBHP -treated NPCs (p< 0.05) Figure (4A, 4B). Interestingly, the same groups showed the highest concentration of the M2 macrophages markers with p< 0.05. That is to say, the levels CD163, Arg-1 and MR were markedly increased when DNMT1was inhibited (Figure 4C–4E), and the levels of iNOS and TNF-α were decreased when DNMT1 was inhibited (p<0.05) Figure (4A, 4B).

Furthermore, western blot analysis showed that the protein expression levels of SIRT6 were significantly increased (p < 0.05) upon inhibiting DNMT1 which indicates the negative regulatory role of DNMT1 on SIRT6 expression as shown in Figure 4F, 4G.

The abovementioned results were consistent with the CD206 and CD86 flow cytometry analyses. CD206 and CD86 are specific markers for M1 and M2 macrophages respectively, which were used to examine the macrophage phenotypes of all groups (Figure 4J).

### DNMT1/SIRT6 axis can affect the pyroptosis of nucleus pulposus cells by regulating macrophage polarization

**Figure 5:**
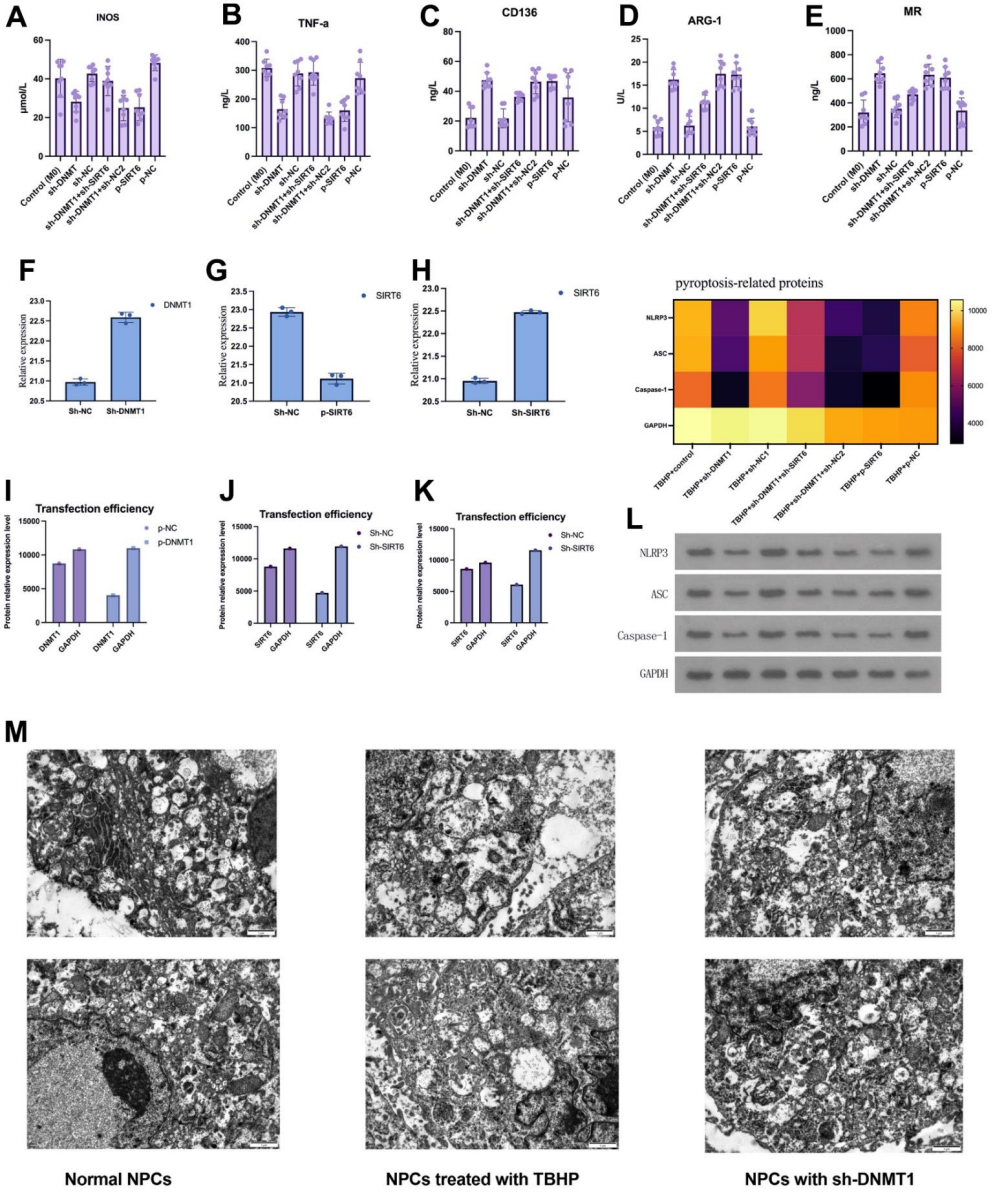
**DNMT1/SIRT6 axis can affect the pyroptosis of nucleus pulposus cells by regulating macrophage polarization.** (**A**–**E**) The expression of M1/M2 macrophage specific markers detected by ELISA. (**F**–**K**) Western blot and quantitative PCR to evaluate the transfection efficiency. (**L**) Heatmap of pyroptosis-related proteins expression which detected by western blot. (**M**) Transmission electron microscopy showed the autophagosomes in NPCs.

Pyroptosis is closely related to inflammation. Herein, we investigated the role of DNMT1/SIRT6 axis in pyroptosis by studying its effect on macrophage polarization. The expression of M1 macrophage iNOS and TNF-α (Figure 5A, 5B), and the expression of M2 macrophage specific markers CD163, Arg-1 and MR were detected by ELISA (Figure 5C–5E). The M1 macrophages markers including iNOS and TNF-a were significantly decreased in the group with inhibited DNMT1 and overexpressed SIRT6 (p< 0.05). On the other hand, the M1 macrophage markers were significantly increased in the group with inhibited DNMT1 and overexpressed SIRT6 (p<0.05). Moreover, pyroptosis-related proteins NLRP3, ASC and caspase-1 were detected by western blotting. Quantification analysis showed that TBHP+sh-DNMT1, TBHP+sh-DNMT1+sh-NC2, and TBHP+p-SIRT6 groups presented the lowest levels of all pyroptosis-related proteins NLRP3, ASC and caspase-1 (p<0.05) (Figure 5L). Additionally, the transfection efficiency was detected by WB/PCR. The transfection efficiency was obviously efficient. When SIRT6 combined with Sh-SIRT6, it presented a low value compared to SIRT6 when combined with p- SIRT6 (Figure 5F–5K). Transmission electron microscope finding revealed that the numbers of autophagosomal vacuoles increased after inhibition of DNMT1 or overexpression of SIRT6, indicating that autophagy was in play.

### DNMT1/SIRT6 axis can affect NPCs proliferation and apoptosis by regulating macrophage polarization

**Figure 6:**
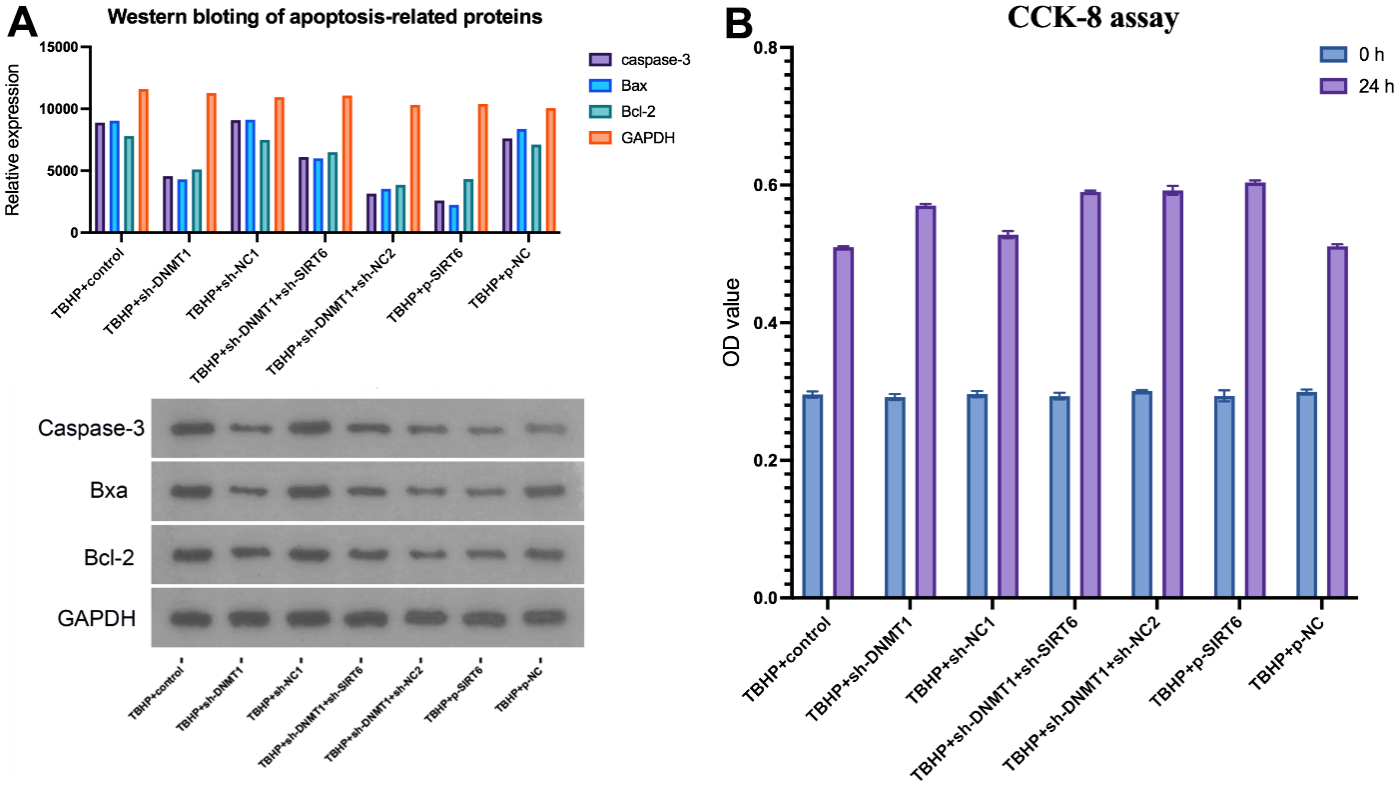
**DNMT1/SIRT6 axis can affect NPCs proliferation and apoptosis by regulating macrophage polarization.** (**A**) apoptosis-related proteins including caspase-3, Bax, and Bcl-2 were detected by western blot (**B**) CCK-8 assay for cell viability.

The CCK-8 assay was used to evaluate NPCs’ proliferation and survival. After transfection, the TBHP + sh-DNMT1 + sh-NC2, TBHP + p-SIRT6, and TBHP + sh-DNMT1 groups showed significantly higher viability rates than the control groups (p<0.05) (Figure 6B). CCK-8 assay results revealed that interference with SIRT6 and sh-DNMT1 promoted the proliferation of NPCs (p<0.05).

Cell apoptosis was detected by flow cytometry, and apoptosis-related proteins, including caspase-3, Bax, and Bcl-2 were detected by western blotting (Figure 6A). TBHP + sh-NC1, TBHP + p-NC, and TBHP + control groups presented the highest apoptosis rate with higher apoptosis-related proteins relative expression compared with other groups (p<0.05).

However, the cells that were transfected with p-SIRT6 or sh-DNMT1 experienced a significant decrease in the apoptosis rate (p<0.05).

## DISCUSSION

IDD has been shown to be associated with an increase in the expression of inflammatory factors, which strongly suggests that the development and progression of IDD is connected to inflammatory response, which is a vital homeostatic event in well-being and disease [13]. Unlike classical phagocytotic “M1 macrophages,” M2-polarized macrophages act as regulators of tissue repair and remodeling [14–16]. Previous studies have shown that DNMT has a causal correlation with chronic inflammation-associated diseases and macrophage polarization [17, 18]. DNMT1 performs a vital role in several processes, such as gene modulation, maintenance of methylation, and chromatin stability [19]. DNMT1 has also been used in several studies as a biomarker of age-related macular degeneration [9, 20].

Inhibition of DNMT1 has been carried out in several areas of research such as in arthritis and ankylosing spondylitis [21, 22]. DNMT1 showed an ability to stimulate M1 macrophage activation, which subsequently promotes the inflammatory response [23–25]. Therefore, the inhibition of macrophage-specific DNMT1 may offer an effective and promising therapeutic potential to reduce or prevent the progression of IDD by suppressing M1 macrophage activation. However, the role of DNMT1 in IDD had not been studied hitherto.

Our study showed that, in the IDD rat model, the expression of DNMT1 was upregulated and, on the contrary, DNMT1 inhibition led to suppression of the progression of degenerative changes in NPCs and nucleus pulposus tissues via suppressing the M1 macrophage polarization and promoting the M2 macrophage polarization. DNMT1 silencing in macrophages (M0) co-cultured with NPCs caused a decrease in the expression of M1 macrophage-specific markers (iNOS and TNF-α), which indicates a reduction in the expression of inflammatory cytokines and promotion of the expression of M2 macrophage-specific markers (CD163, Arg-1, and MR), which could subsequently delay the progression of IDD changes.

On the other hand, sirtuins play a pivotal role in the regulation of inflammatory responses [26]. SIRT6 exerts its effects in various cellular processes such as apoptosis, inflammation, and aging [27, 28]. Several studies have showed the therapeutic effect of SIRT6 overexpression such as protecting cardiomyocytes from apoptosis/necrosis and ameliorating osteoarthritis through inhibiting extracellular matrix degeneration and cellular senescence [29–31]. Moreover, studies have also showed that SIRT6 deficiency triggering macrophage polarization toward the M1 phenotype and vice versa [32–34].

Herein, SIRT6 expression in the IDD model group was downregulated. SIRT6 overexpression groups showed a suppressed M1 macrophage polarization and promoted M2 macrophage polarization, which led to decrease the progression of degenerative changes in NPCs and nucleus pulposus tissues. Contrary to this, the experimental group which experienced decreased SIRT6 expression presented a promoted M1 polarization, inhibited M2 polarization, and increased proinflammatory agent secretion. This finding was in keeping with previous studies which revealed SIRT6 inhibition in an osteoarthritis model [33]. Moreover, in the SIRT6-deficient mice, the degenerative phenotype was promoted and the mice experienced premature aging traits including, but not limited to, kyphosis, and osteopenia which could be explained as a failure in base excision repair [35, 36].

Furthermore, the role of SIRT6 on IDD had been investigated previously [28]. The study results showed that SIRT6 reduced apoptosis and senescence by promoting autophagy that eventually ameliorated the IDD [28]. These findings are consistent with our study. The experimental group which experienced SIRT6 overexpression showed an inhibition of M1 macrophage activity and enhanced M2 macrophage polarization, besides suppression of apoptosis. Together, the experimental findings showed that inhibition of DNMT1 or overexpression of SIRT6 suppresses M1 polarization and promotes M2 polarization of macrophages.

The ability to regulate pyroptosis and apoptosis makes our proposed approach promising. Pyroptosis is a programmed cell death process that promotes local inflammatory responses and plays a significant role in pathogenesis of IDD [37]. Several studies have shown the association between M1 polarized macrophages and pyroptosis [38–40]. Herein, inhibition of DNMT1 or overexpression of SIRT6 leads to suppressed M1 polarization and subsequently inhabitation of pyroptosis.

Moreover, the degenerative changes observed in IDD have also been linked to NPCs apoptosis [41–44]. Zi et al., in their study showed that Sirt6-induced autophagy inhibited pyroptosis, and the downregulation of Sirt6 was associated with poor prognosis and serious endothelial damage in acute myocardial infarction patients [45]. Interestingly, DNMT1 silencing or overexpression of SIRT6 inhibit pyroptosis and apoptosis, which could be a potential therapeutic target because of its protective effects.

The performed investigations showed that DNMT1 silencing negatively regulated the expression of SIRT6, suggesting that inhibition of DNMT1 could have dual effects on both inhibition of DNMT1 expression and promotion of SIRT6 expression. Furthermore, this study revealed another benefit of inhibiting DNMT1 and promoting expression of SIRT6, which is promoting cells viability and decreasing the apoptosis rate through regulating macrophage polarization toward M2 polarization.

The limitations of the current study encompass a small sample size, absence of clinical data and clinical sample analysis, as well as incomplete investigation of SIRT6-related signaling pathways.

Overall, the current *in-vitro* and *in-vivo* study showed that inhibition of DNMT1 eased IDD changes through suppression of the inflammatory response. The inhibition of DNMT1 or overexpression of SIRT6 induced the “M2” polarization of macrophages. Moreover, the expression of SIRT6 can be promoted through inhibition of DNMT1 which showed a regulatory role over SIRT6. Finally, inhibition of DNMT1 or overexpression of SIRT6 showed the ability to promote cell viability, and inhibits pyroptosis and apoptosis, which all together offer a promising method to delay and suppress degenerative changes.
